# *Gagea kotuchovii* (Liliaceae) a new species from the Karatau Mountains (western Tian Shan, Kazakhstan) evidenced by morphological and molecular analyses

**DOI:** 10.1371/journal.pone.0336223

**Published:** 2025-12-05

**Authors:** Serik Kubentayev, Igor Levichev, Shukherdorj Baasanmunkh, Ewelina Klichowska, Daniyar Alibekov, Hyeok Jae Choi, Marcin Nobis

**Affiliations:** 1 Astana Botanical Garden, Astana, Kazakhstan; 2 Komarov Botanical Institute of RAS, Saint Petersburg, Russia; 3 Department of Biology and Microbiology, Changwon National University, Changwon, Korea; 4 Institute of Botany, Faculty of Biology, Jagiellonian University, Kraków, Poland; Zhejiang Agriculture and Forestry University: Zhejiang A and F University, CHINA

## Abstract

*Gagea kotuchovii* (Liliaceae) a new narrow endemic species growing exclusively in the Karatau Mountains in South Kazakhstan, is here described and illustrated. It is a unique species within the section *Gagea*, differing from closely related taxa by the presence of several stolons of different lengths (0.5–8 cm), formed on a single vegetative individual, as well as a unique bulb with a densely woolly sheath. Morphological characteristics, distribution map, and illustrations of the habit and habitats of the new species are presented. We also present phylogenetic analyses based on the internal transcribed spacer (ITS) of nuclear DNA and SNPs obtained from DArT genome-wide sequencing (Diversity Arrays Technology sequencing), which confirmed the isolated position of *G. kotuchovii* but also revealed its phylogenetic relation with morphologically similar species. Additionally, our results reveal phylogenetic intergeneric organization of *Gagea* representatives occurring in Kazakhstan and/or Middle Asia. To facilitate morphological identification of Asian *Gagea* with stolons, and similar to *G. kotuchovii*, we present an identification key.

## Introduction

The genus *Gagea* Salisb. (star-of-Bethlehem), distributed throughout Eurasia and along the Mediterranean coast of Africa, includes over 330 species, making it the largest and most taxonomically complex genus in the Liliaceae family [1]. The highest diversity of *Gagea* species is distributed in the mountains of Pamir-Alai and Western Tian Shan, with 122 and 77 species, respectively [1–3]. The genus *Gagea* significantly contributes to the remarkable species richness of the Mountains of Central Asia, one of the world’s 36 biodiversity hotspots [4,5]. Morphologically, *Gagea* differs primarily in its small, grass-like leaves, yellow flowers, early spring blooming period, and the presence of a fibrous-coated bulb**,** distinguishing it from other Liliaceae members such as *Tulipa*, *Fritillaria*, and *Lilium***,** which have broader leaves and larger, more conspicuous flowers [6,7]. The members of this genus exhibit pronounced morphological variability in vegetative and generative organs at different stages of ontogenesis, mainly due to the influence of various environmental conditions [2,6].

The flora of Kazakhstan is represented by 63 species of *Gagea*, with an uneven distribution across the country [8] of which only four species are considered national endemics [9]. The genus *Gagea* is also one of the most numerous and at the same time most problematic genera in Kazakhstan and Middle Asia. Many *Gagea* species still need taxonomic revision using integrative, morphological and molecular approaches. For instance, recent integrative analyses on ecology, morphology and phylogenetic relationships reveal that *G. altaica* and *G. sarysuensis*, described solely based on morphology, are fully conspecific with *G. alberti* Regel [10]. On the other hand, many new species were recently described, and it is estimated that only in the Mountains of the Middle Asia, ca 20% of species are still undescribed [11–15]. As a result of our extensive field studies associated with a collection of herbarium specimens and detailed photographs of *Gagea* from the various vegetation types, we found in the Turkestan region (Karatau Mts, NW Tian Shan), several individuals of *Gagea* sp. which are morphologically most similar to *G. brevistolonifera* Levichev and *G. turkestanica* Pascher. However, based on revision of the relevant literature [16–18] and herbarium specimens stored at LE, MW, TASH, AA, and NUR [19], we concluded that the collected specimen may represent a new, undescribed species. According to morphological characters of peduncle structure, basal leaf, inflorescence shape and bulb shape, the newly discovered species should be assigned to the section *Gagea*. It includes about 60 species of *Gagea* [7], characterized by few-flowered umbellate inflorescence, with a whorl of few leaves (mostly only 2) at the basis; peduncle in cross-section is 4–5 sided, and single basal leaf, in cross-section angular with one or rarely three keels at the lower surface, narrow-lineal or, less often, broad-lineal, of bifacial type, whereas the second leaf is always connate with the peduncle almost up to the basis of the inflorescence; tepal apex is rotund or obtuse, capsule trigonous or broadly triangular, shorter than the persistent tepals, and terete seeds [6,7].

One of the reliable characters for the separation of species within the section *Gagea* is the features of the vegetative bulb structure, as well as the type of vegetative reproduction [1,2,20,21]. The putative new species attracted attention by the structure of the vegetative bulb, on which several stolons of different lengths are formed, each of which bears a single bulb. This phenomenon has not been observed in the section *Gagea* before.

This paper aims to describe a new species of *Gagea* from Kazakhstan based on extensive morphological analysis and provide its phylogenetic relation to the most morphologically similar species of *Gagea* occurring in Kazakhstan and Middle Asia.

## Materials and methods

### Ethics statement

Attached to this manuscript is a completed questionnaire that outlines the ethical, cultural, and scientific considerations characteristic of inclusivity in global research (Supporting Information Inclusivity in global research). Field collection was conducted in accordance with local regulations. The collection site is not legally protected, and the species is not listed as endangered. Therefore, no permits were required.

### Taxon sampling and field expeditions

The present study is based on material collected in the Karatau Mountains of Turkestan Province, South Kazakhstan. The putative new species, here and below named as *Gagea kotuchovii* was first found by the first author in early April 2024 during fieldwork in the Karatau area; at the time of discovery, the plants had already finished flowering. The population was re-surveyed at the end of March 2025, when it was possible to record flowering individuals and collect the complete morphological material needed to describe the new species. During the expedition, 12 flowering specimens and about 20 vegetative plants were collected. All morphological measurements were made on living plants using a standard measuring ruler. The background in the distribution map (Fig 6) has been adapted from the United States Geological Survey (USGS; https://www.usgs.gov; public domain) [22].

**Fig 1:**
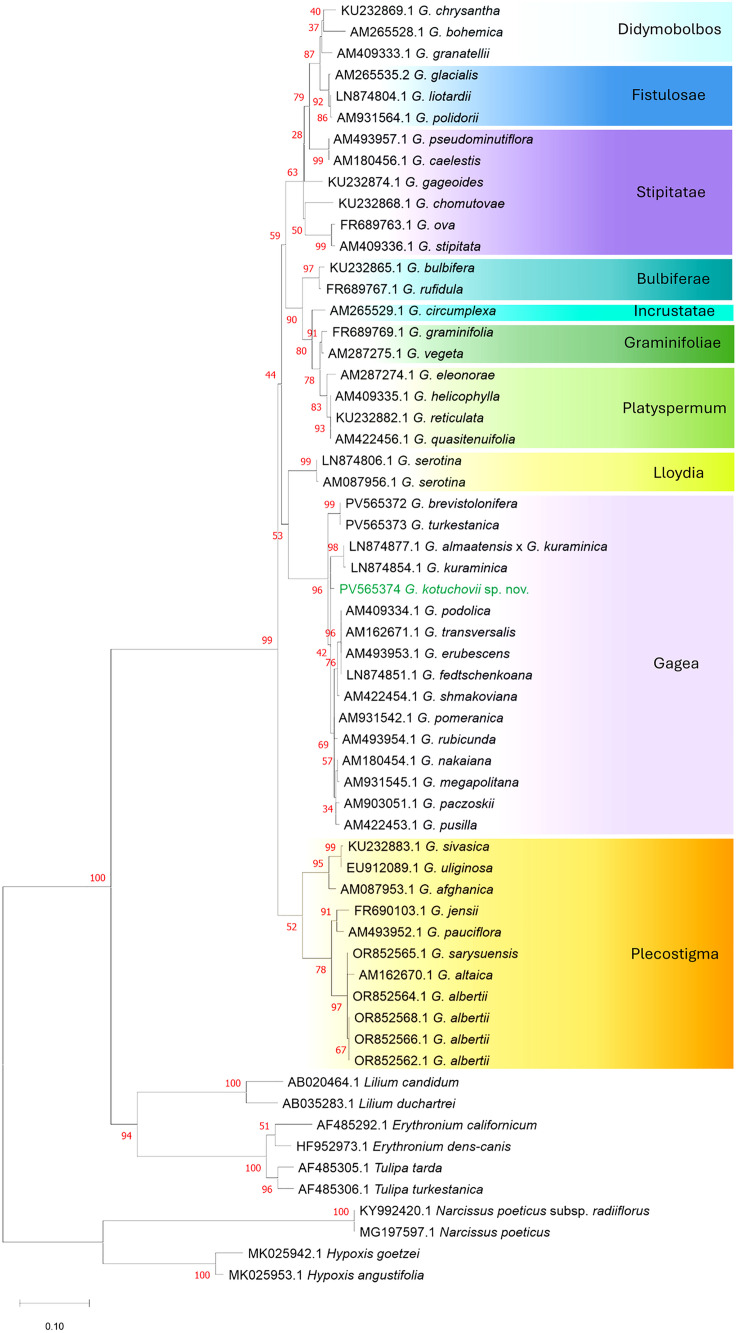
The Maximum Likelihood phylogenetic tree of the genus *Gagea* based on 498 bp of internal transcribed spacer (ITS) with bootstrap values (in red). The analysis involved 50 *Gagea* individuals and 10 individuals from different genera within the Magnoliopsida class as an outgroup. The scale bar refers to substitutions per site phylogenetic distance.

**Fig 2:**
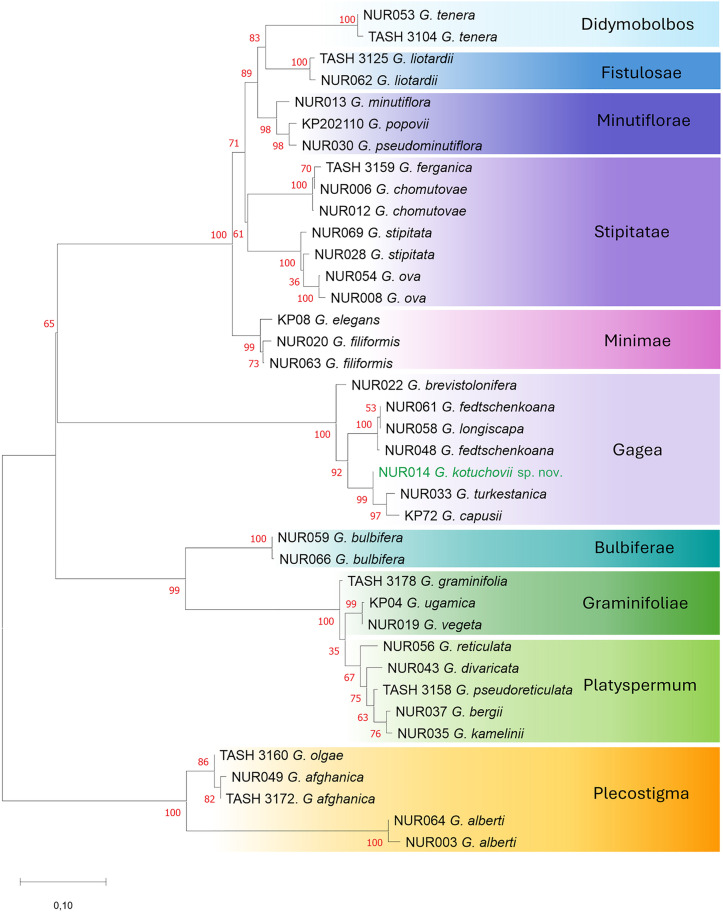
The Maximum Likelihood phylogenetic tree of the genus *Gagea* based on 1,199 DArTseq-derived SNPs with bootstrap values (in red). The analysis involved 39 *Gagea* individuals, including five individuals of the section *Plecostigma* as an outgroup. The scale bar refers to substitutions per site phylogenetic distance.

**Fig 3:**
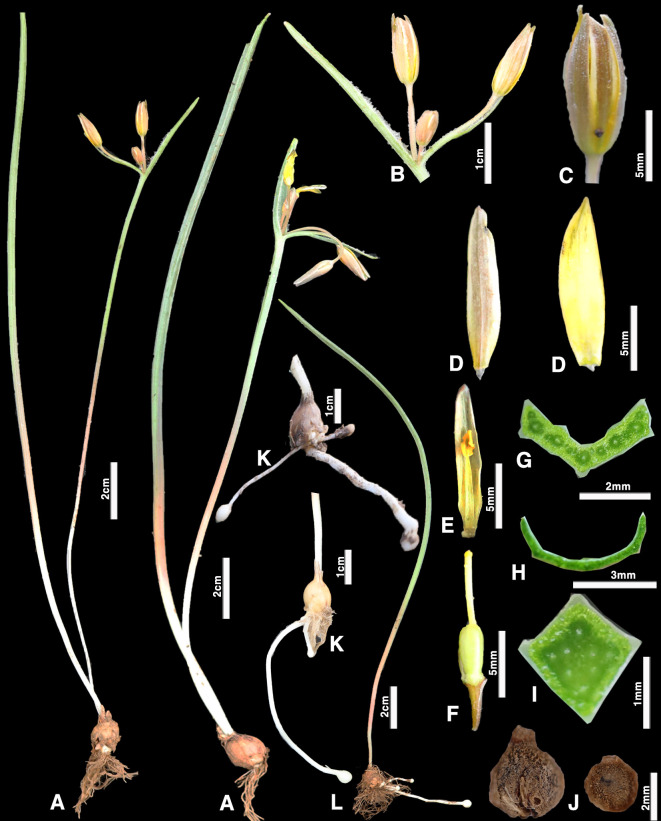
*Gagea kotuchovii*; (A) General habit; (B) inflorescence; (C) flower; (D) outer petal of the perianth; (E) inner petal of the perianth (F) perianth petal with a stamen; (G) gynoecium; (H) basal leaf transverse section; (J) peduncle cross-section; (I) stem leaf transverse section; (K) last year’s juvenile bulb; (L) bulb of a vegetative individual with several stolons; (M) vegetative individual (Photo by: S. Kubentayev).

**Fig 4:**
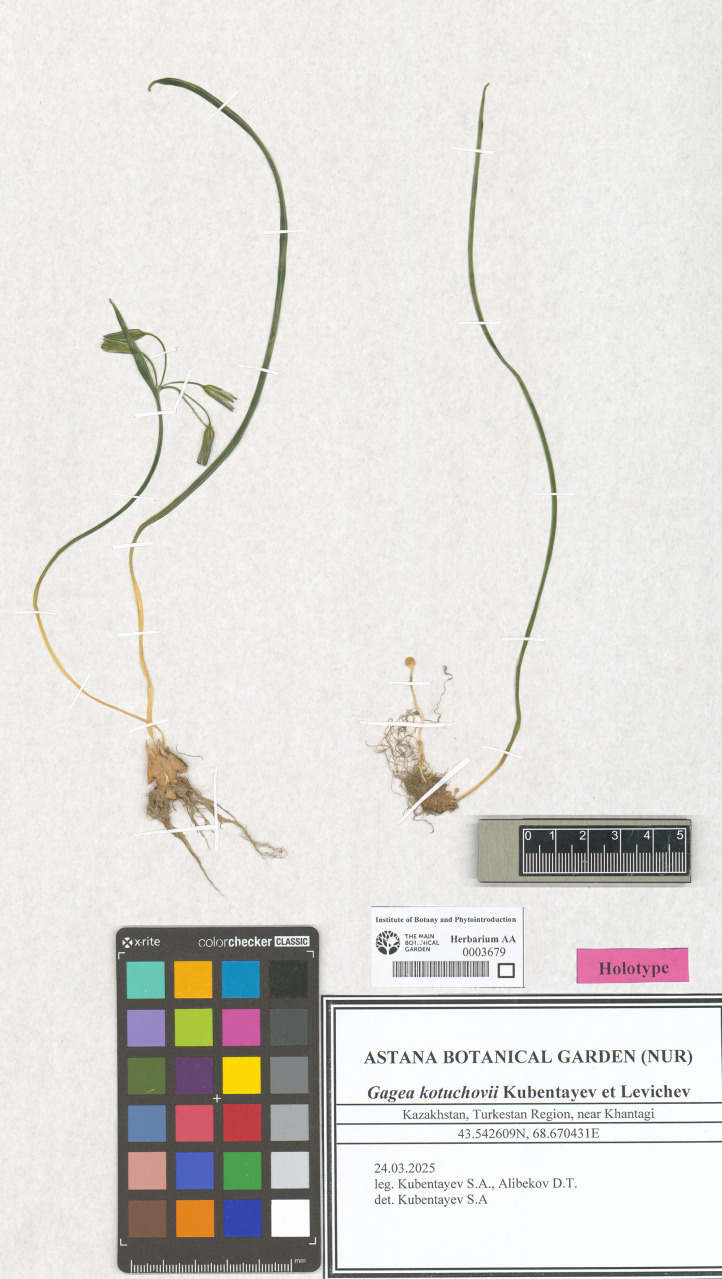
Holotype of *Gagea kotuchovii* preserved in the AA herbarium.

**Fig 5:**
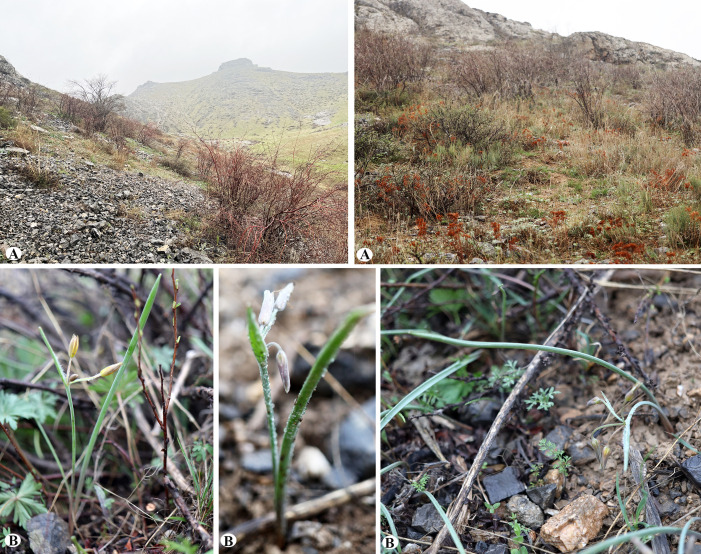
Habitats of *Gagea kotuchovii *(A); General habit of the species (B) (Photo by: S. Kubentayev).

### DArT analysis: DNA extraction, genomic library preparation, genome complexity reduction-based sequencing, and data filtration

This section was performed according to previously published procedures [23,24]. Genomic DNA was extracted using the Genomic Mini AX Plant kit (A&A Biotechnology). Initially, dried leaf tissue was ground to a fine powder using a mixer mill MM400 (Retsch) and 3–5 mm glass beads. The NanoDrop ND1000 spectrophotometer (Thermo Fisher Scientific) was used to assess the purity and concentration. To meet the DArTseq genotyping protocol requirements, the concentration of each sample was adjusted to 50–100 ng/μL. All DNA samples were processed in digestion/ligation reactions as described by Kilian et al. [25], but replacing a single PstI-compatible adaptor with two different adaptors corresponding to two different restriction enzyme overhangs. The PstI-compatible adapter was designed to include Illumina flowcell attachment sequence, sequencing primer sequence, and “staggered. The used reverse adapter included a flowcell attachment region and MseI-compatible overhang sequence. Only “mixed fragments” (PstI-MseI) were effectively amplified by PCR using an initial denaturation step of 94°C for 1 min, followed by 30 cycles with the following temperature profile: denaturation at 94°C for 20 s, annealing at 58°C for 30 s and extension at 72°C for 45 s, with an additional final extension at 72°C for 7 min. Equimolar amounts of PCR amplification products from each of the 96 samples from the microtiter plate were bulked and applied to c-Bot (Illumina, USA) bridge PCR. Then the single read sequencing on Hiseq2500 (Illumina, USA) for 77 cycles was performed. Next, proprietary DArT analytical pipeline was used to process sequences generated from each lane. The poor-quality sequences were filtered away from fastq files, with more stringent selection criteria for the barcode region than for the rest of the sequence. This ensured reliable assignment of sequences and specific samples during the “barcode split” were reliable. During the marker calling step, ca. 2.5 million sequences per barcode/sample were identified.

We applied codominant single-nucleotide polymorphism (SNP) markers processed in R v. 4.0.3 [26] with the additional dartR v.2 package [27]. From the obtained 5,514 SNPs we removed all monomorphic loci. Due to distant phylogenetic relationships among analyzed species, we used a low threshold (0.35) for the loci call rate. We filtered loci with a scoring reproducibility lower than 100% and secondary SNPs (randomly selecting one per allele ID). We removed SNPs with a minor allele frequency <0.05. Finally, for phylogenetic analyses purposes, the remaining 1,199 SNP bases were concatenated across loci to generate a single combined sequence. Heterozygous positions were replaced by the standard ambiguity codes.

### ITS analysis: DNA extraction, amplification, and sequencing

The sequences of the internal transcribed spacer (ITS) for three individuals of *Gagea* were obtained from individuals we collected (a list of localities and individuals is presented in Supporting Information S1 Table). The modified CTAB method used for DNA extraction and Sanger sequencing is described in our previous study [10]. Nucleotide sequences of *G. brevistolonifera* Levichev, *G. turkestanica* Pascher, and *G. kotuchovii* Kubentayev et Levichev *sp. nov.* were deposited in the National Center for Biotechnology Information (NCBI) under accession numbers PV565372, PV565373, and PV565374, respectively. The remaining 47 sequences of the genus *Gagea* and 10 sequences from individuals belonging to closely related genera (as outgroups) were downloaded from the NCBI GenBank database (GenBank 2024) (accession numbers are included in Fig 8). ClustalW Multiple Alignment from BioEdit v. 7.2.6.1 [28] was used to align the ITS sequences. The obtained alignment has a length of 498 bp.

**Fig 6:**
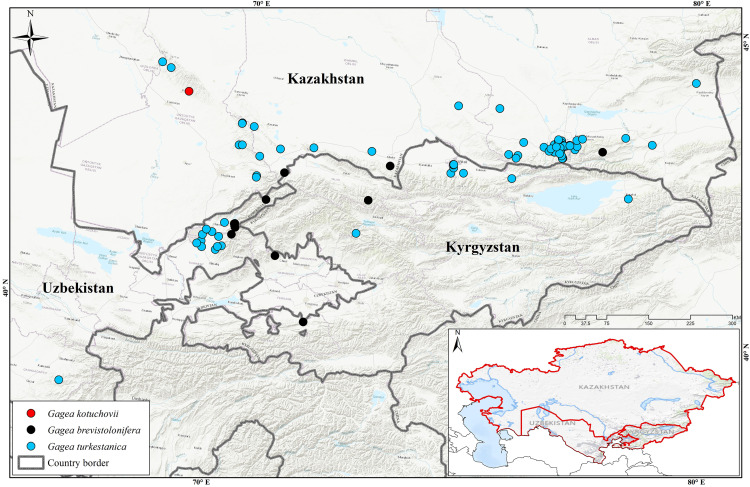
Distribution map of *Gagea kotuchovii*, *G. turkestanica*, and *G. brevistolonifera* in Middle Asia; the map background has been adapted from the United States Geological Survey (USGS; https://www.usgs.gov; public domain).

**Fig 7:**
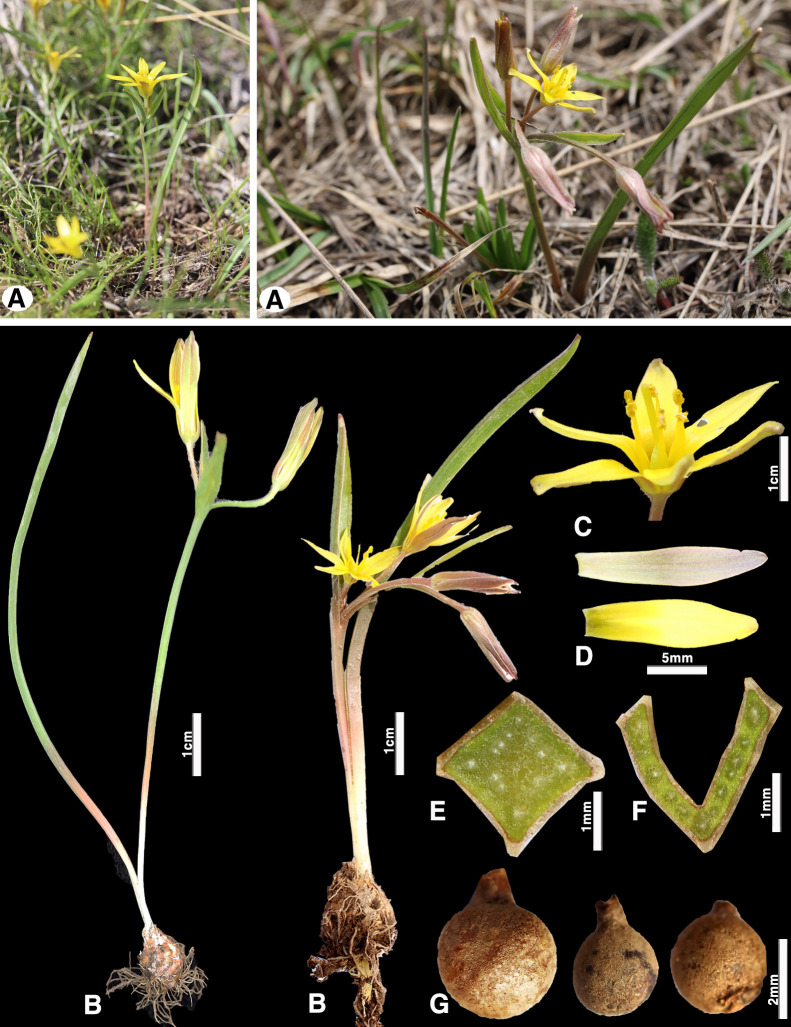
*Gagea turkestanica*; (A) general habit; (B) general appearance;(C) flower; (D) Outer and inner petals of the perianth; (E) peduncle cross-section; (F) basal leaf transverse section; (G) last year’s juvenile bulb (Photo by: S. Kubentayev).

**Fig 8:**
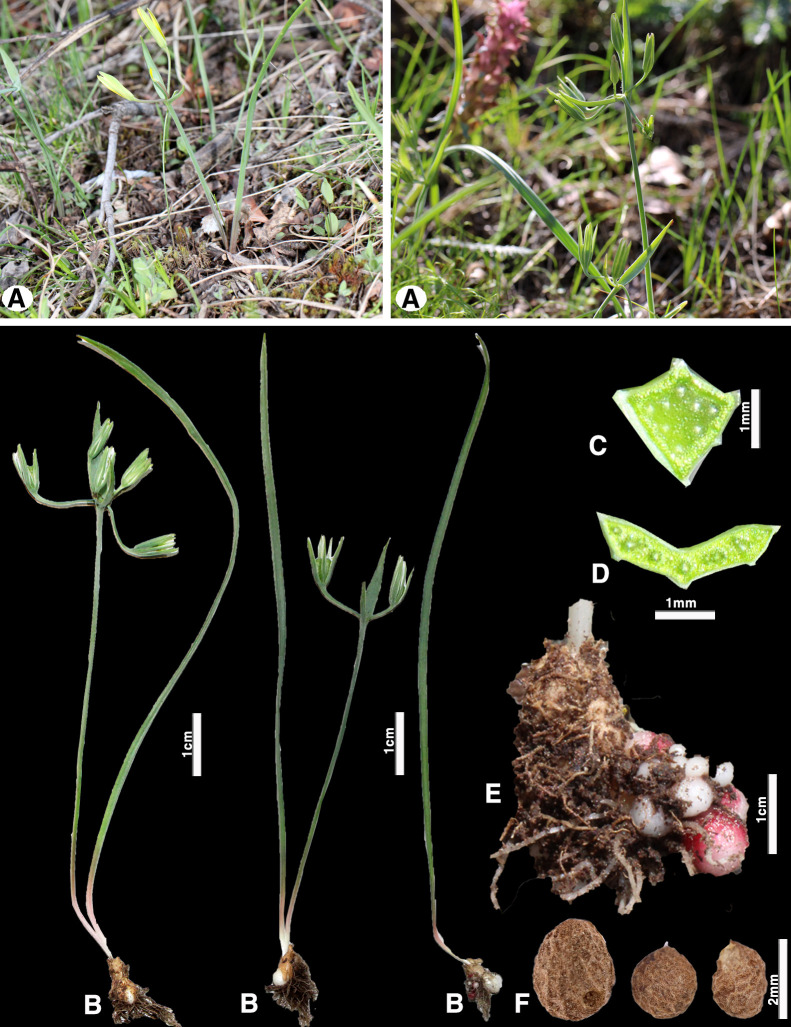
*Gagea brevistolonoifera*; (A) general habit; (B) general appearance; (C) peduncle cross-section; (D) basal leaf transverse section; (E) vegetative bulb with a short stolon and a group of small bulbs; (F) last year’s juvenile bulb (Photo by: S. Kubentayev).

### Analysis of phylogenetic relationships

The phylogenetic relationships of the studied *Gagea* representatives were inferred by using the Maximum Likelihood phylogenetic trees constructed with MEGA version 11.0.13 [29]. In the case of the ITS-based analysis, including 50 individuals of *Gagea* and 10 individuals of distantly related genera as an outgroup (class Magnoliopsida), the Tamura-Nei model with gamma-distributed rate variation across sites and a proportion of invariable sites was chosen as the best-fit substitution model based on the Akaike information criterion (AIC) values. Whereas in the case of DArTseq-based analysis performed for 39 individuals of the genus *Gagea,* the Kimura 2-parameter model with uniform rates among sites was chosen (Fig 8). Based on the result of ITS phylogenetic tree, which indicated that the *Plecostigma* section is sister to the other *Gagea* members included in the analysis, in the case of the DArTseq-based tree, we used *Plecostigma* to root the tree. For both analyses, we used the bootstrap method with 1,000 replications to assess the reliability of phylogenetic trees.

The SNP dataset derived from the DArTseq pipeline in the genlight format, as well as alignments from ITS and SNPs in the fasta format, are available via the Figshare repository: https://doi.org/10.6084/m9.figshare.29626295.v2.

### Conservation assessment

The conservation status of *G. kotuchovii* was assessed by the Geospatial Conservation Assessment Tool [GeoCAT; https://geocat.iucnredlist.org/, 30] based on a 2 km^2^ grid cell size (IUCN 2012). This tool performs rapid geospatial analysis based on georeferenced data and automatically evaluates the conservation status solely based on the extent of occurrence (EOO) and area of occupancy (AOO) values [30]. The categories and criteria of threatened species were defined according to IUCN Standards and Petitions Committee [31].

#### Nomenclature.

The electronic version of this article in Portable Document Format (PDF)

in a work with an ISSN or ISBN will represent a published work according to the International Code of Nomenclature for algae, fungi, and plants, and hence the new names contained in the electronic publication of a PLOS ONE article are effectively published under that Code from the electronic edition alone, so there is no longer any need to provide printed copies.

In addition, new name contained in this work have been submitted to IPNI, from where

they will be made available to the Global Names Index. The IPNI LSIDs can be resolved and the associated information viewed through any standard web browser by appending the LSID contained in this publication to the prefix http://ipni.org/. The online version of this work is archived and available from the following digital repositories: PubMed Central, LOCKSS.

#### Inclusivity in global research.

Additional information regarding the ethical, cultural, and scientific considerations specific to inclusivity in global research is included in the Supporting Information Inclusivity in global research.

## Results

### Molecular analyses

The Maximum Likelihood phylogenetic trees revealed that examined specimens of *Gagea* formed several different clades corresponding with the intrageneric sectional division of the genus, based on morphological characters (Figs 1, 2). In both phylogenetic analyses, the new species, described below as *Gagea kotuchovii*, is located within the clade comprising species from the nominal *Gagea* section, however, the new taxon is clearly separated from all the examined taxa. The phylogenetic tree based on 498 bp of internal transcribed spacer (ITS) involved 50 *Gagea* individuals of 46 species, with 23 of them occurring in Kazakhstan. The species represent 10 sections, with section *Plecostigma* as the most external and sister to all the other (Fig 1). Based on ITS analysis, *G. kotuchovii* is an independent taxon representing the nominal *Gagea* section, well separated from morphologically most similar *G. turkestanica* and *G. brevistolonifera*, which were clustered in the common subclade (Fig 1), as well as from *G. kuraminica* Levichev and all the remaining taxa representing the section *Gagea*, organized in sister, but polytomic subclade (Fig 1). A similar phylogenetic relationship was revealed by the tree based on 1,199 SNPs (Fig 2), and comprising 39 individuals of 29 species. Although nominal *Gagea* sect. *Gagea* is represented here by a lower number of representatives; the clade is well-resolved and supported by a high bootstrap value. However, *Gagea kotuchovii* together with *G. turkestanica* and *G. capusii* A.Terracc. are grouped in a common subclade which is here a sister to the one containing *G. fedtschenkoana* Pascher and *G. longiscapa* Grossh. Whereas, in contrast to ITS tree, *G. brevistolonifera* is separated from all the above mentioned taxa, and located in SNP-based tree as the most external within the *Gagea* clade (Fig 2).

### Taxonomic treatment

***Gagea kotuchovii* Kubentayev et Levichev, sp. nov.** (Figs 3, 4, Supporting Information S1 Fig)

[urn:lsid:ipni.org:names:77371301-1]

Type: Kazakhstan, Turkestan Region, near Khantagi, 43.542609 N, 68.670431 E, 24 March 2025, *S.A. Kubentayev and D.T. Alibekov* s.n. (holotype AA0003679!, isotype NUR, LE).

Diagnosis. From all species of the nominal *Gagea* section, including the most similar *G. turkestanica* and *G. brevistolonifera*, the new species is easily distinguished by the presence of a group of stolons of different length on bulbs of vegetative individuals (Fig 3L, 3M), ending in single bulbs, which, after overwintering, are covered with densely woolly growths (Fig 3K).

Description. Plants slender, solitary, 8–20 cm in height (Fig 3A). Bulb ovoid, 8–12 mm in diameter, covered with light brown, leathery tunics. Juvenile specimens form 2–10 stolons of varying lengths (0.5–8 cm) (Fig 3L, M), each ending in a single bulb. Bulbs white during development and turn dark brown after winter dormancy due to a dense layer of short papillate (Fig 3K); occasionally, a sessile (flattened) bulb may be present, not develop a stolon and thus bear an attachment scar (Fig 3K). Scape 6–15 cm tall, quadrangular in cross-section (Fig 3J). Basal leaf solitary, 4–6 mm wide, linear-obovate, angular in cross-section (Fig 3H), exceeds the inflorescence by one-third. Two subtending leaves present, oppositely arranged, 2–4 cm long; the lower one equal to or slightly exceeds the length of the inflorescence, boat-shaped in cross-section (Fig 3I) with two ridges on the underside. Inflorescence remains an umbel in shape, 3–5-flowered (Fig 3B). Pedicels unequal, 1.5–3 cm long, curve downward after flowering. Tepals (Fig 3D, E) lanceolate, obtuse, 10–13 mm long and 3–6 mm wide; yellow on the inner surface, greenish with light margins on the outer surface; the inner tepals shorter and narrower than the outer ones. The filaments (Fig 3F) linear, reaching half the length of the tepals. Anthers yellow. Ovary elongate, sessile (Fig 3G). Style with a small stigma, shorter than the tepals. Capsule inversely ovoid, sessile. Seeds cylindrical.

Habitat ecology. G*agea kotuchovii* grows on stony-clay soils among shrubs at an altitude of 620 m a.s.l., on the debris of slopes in the ravines with northern and northeastern exposures of the Karatau Mts (Fig 5). The following species are often found in the community with *G. kotuchovii*: *Cerasus erythrocarpa* Newsky, *Cotoneaster allochrous* Pojark., *Rosa persica* Michx. ex Juss., *Ephedra intermedia* Schrenk & C.A.Mey., *Artemisia karatavica* Krasch. & Abolin ex Poljakov, *Sibbaldianthe orientalis* (Juz. ex Soják) Mosyakin & Shiyan, *Schrenkia involucrata* Regel & Schmalh., *Ferula tenuisecta* Korovin, *Tulipa orthopoda* Vved., *Delphinium longipedunculatum* Regel & Schmalh., *Geranium linearilobum* DC., *Corydalis sewerzowii* Regel, *Fritillaria stenanthera* (Regel) Regel, *Allium inconspicuum* Vved.

Distribution. Endemic to the Karatau Mountains in South Kazakhstan. Currently, it is known only from Khantagi (Fig 6).

Etymology. The new species is named in honour of the eminent florist and taxonomist Yuri Andreevich Kotukhov (lat. J.A. Kotuchov), who greatly contributed to the study of the flora of Kazakhstan and surrounding regions.

Similar species: *Gagea turkestanica* Pascher (Fig 7) was described from the vicinity of Almaty (formerly Verny) in Kazakhstan and grows on gentle slopes and plains in foothill and lowland areas. This species is one of the most widespread and frequently noted members of the section *Gagea* in Central Asia (Fig 6). *Gagea turkestanica* differs from *G. kotuchovii* by the absence of several stolons on the vegetative bulb, and by the light-grey leathery (not woolly) tunic of the overwintering bulb. According to Plants of the World Online [32] and other botanical databases, *G. turkestanica* is considered a synonym of *G. capusii*, however, we believe that *G. turkestanica* should be treated as a distinct taxon. Because *G. capusii* differs from *G. turkestanica* in the structure of the basal leaf: the former has a grooved-triangular, fistulous cross-section, while the latter has an angular and non-fistulous basal leaf [17]. Additionally, *G. capusii* develops vegetative bulbs in both vegetative and generative individuals, whereas in *G. turkestanica*, bulbils are formed only in vegetative plants.

*Gagea kotuchovii* is also slightly similar to *G. brevistolonifera* (Fig 8) which differs by the formation of clusters of vegetative bulbils on a short stolon, as well as by its characteristic basal leaf — linear-obcuneate, angular, with a grooved-angular transverse section. The distribution of this species is not well studied, and according to our data, its range does not extend beyond the Western Tien Shan (Fig 6). It was first recorded in the Kyrgyz Alatau and Turgen Gorge of Kazakhstan [33]. Interestingly, the occurrence of this species was mentioned in the Altai Mountains in Mongolia [34], however, this record requires further verification as it is significantly east of the species’ main range.

## Discussion

The Mountains of Central Asia, characterized by highly heterogeneous and isolated habitats with diverse topographies, soil types, and microclimates relating to altitude, slope exposure, and precipitation also known as a biodiversity hotspot, are one of the world’s major centres of plant diversity [4]. Unfortunately, the region is also one of the most sensitive to climate change and biodiversity loss [35–39].

Among the plant genera contributing most to this biodiversity hotspot is *Gagea*, represented by over 100 species [40,41]. In the nominal section of the genus *Gagea*, the new species, *G. kotuchovii*, belongs to a well-defined Central Asian species-group, characterized by dense leathery brown bulb tunics, an umbelliform inflorescence with few flowers and two opposite subtending leaves, as well as a linear, angular in cross-section basal leaf. Besides the mentioned above species, the group includes *G. capusii*, *G. turkestanica*, and *G. brevistolonifera*. Representatives of this group share a common type of vegetative reproduction: vegetative specimens (and in *G. capusii* also generative ones) form a spherical vegetative bulb on a stolon annually [17]. All of these species are also clustered together within the nominal *Gagea* section in both of our molecular analyses (based on ITS and on SNPs), however, in the ITS analysis, *G. kotuchovii* is located independently from *G. turkestanica* and *G. brevistolonifera* grouped in the common subclade. Whereas in the phylogenetic analysis based on SNPs, *G. brevistolonifera* appears to be phylogenetically more distant and located independently from clustered together *G. kotuchovii, G. capusii*, and *G. turkestanica*. These contrasting results between ITS and SNP-based phylogenetic trees may indicate that, despite the morphological similarity of *G. turkestanica* and *G. brevistolonifera*, the two taxa may not be as closely related as they seemed. Whereas, since SNP-based phylogenetic analyses clustered G. fedtschenkoana and G. longiscapa within a common clade, a taxonomic revision of these species occurring in Kazakhstan is needed. Possibly, the morphological variability of *G. fedtschenkoana* is much wider, and in this light, the occurrence of true *G. longiscapa* in Kazakhstan needs confirmation.

In contrast to other species in the group, *G. kotuchovii* exhibits a distinct form of vegetative reproduction, which results in the formation of multiple stolons of varying lengths on a single vegetative specimen, with single bulbs developing at the ends of these stolons. Occasionally, a sessile (flattened) bulb may also be present, which has not developed a stolon, thus bearing an attachment scar. The shape, texture, and coloration of the juvenile bulb tunics are considered important morphological traits with significant taxonomic value when differentiating species in the genus *Gagea* [1,21]. The overwintered juvenile bulbs of *G. kotuchovii* are characterized by a tunic covered with dense, hair-like outgrowths, which is a unique morphological feature previously undocumented in the genus *Gagea*.

Despite sharing a common type of vegetative reproduction (stolon formation), each species within this group exhibits its own morphological peculiarities in this process. In general, taxa with a similar set of characteristics can be roughly divided into the following groups within the extensive *Gagea* section:

1. Species with one or several (e.g., *G. kotuchovii*) long stolons with a single bulb: *G. kotuchovii*, *G. praemixta* Vved., *G. stolonifera* Popov & Czugaeva (nom. nud.).
2. Species with one long stolon with a cluster of bulbs: *G. calyptrifolia* Levichev, *G. angelae* Levichev & Schnittler.
3. Species with a short stolon and a cluster of bulbs: *G. longiscapa* Grossh., *G. brevistolonifera*, *G. shmakoviana* Levichev, *G. xiphoidea* Levichev.
4. Species with a short stolon and a single bulb: *G. capusii*, *G. ancestralis* Levichev, *G. almaatensis* Levichev, A. Peterson & J. Peterson.
5. Species with a very short stolon and a single bulb: *G. fedtschenkoana*, *G. turkestanica*, *G. kuraminica*, *G. huochengensis* Levichev, *G. tichomirovii* Levichev (nom. nud.).

It should be noted that in *Gagea*, the formation of multiple stolons on the vegetative bulb is also observed in some representatives of other sections — *Gramifoliae* (*G. sarmentosa* K.Koch, *G. ludmilae* Levichev, and *G. nabievii* Levichev) [18] and *Plecostigma* (Turcz.) (*G. chinensis* Y.Z.Zhao & L.Q.Zhao). *Gagea kotuchovii* differs from these species by sectional characteristics. For example, it differs from *G. sarmentosa* K.Koch by having one basal leaf (instead of two: the first — tubular, and the second — flat-concave); from *G. nabievii* Levichev by the angular, flat-grooved shape of the basal leaf (instead of flat and grass-like); from *G. ludmilae* Levichev by a four-angled scape (instead of round); and from *G. chinensis* Y.Z.Zhao & L.Q.Zhao by an umbellate inflorescence and a capitate stigma (instead of a whorled inflorescence and deeply three-lobed stigma).

Although the above-mentioned species belong to other sections and differ from *Gagea kotuchovii* by sectional characteristics, we have included them in the identification key presented below as they share a common feature with the described species — the formation of multiple stolons. Moreover, most of the mentioned above species examined by us molecularly (ITS and wide-genome analyses based on SNPs), were clustered within separate clades corresponding with particular sections distinguished based on morphological characters, which additionally supports our results, and reveals the phylogenetic relations between and within particular clades, although, the species-set differs somewhat in both analyses.

Currently, no comprehensive identification key exists for species of the genus *Gagea.* This is primarily due to the fact that most existing keys are based predominantly on the morphology of above-ground organs, which exhibit a high degree of similarity among species [2,21]. While important traits, such as the type of vegetative reproduction, the morphology of underground organs, and the cross-sectional shape of the basal leaf, have been significantly neglected. Below, we present an identification key for species related to *Gagea kotuchovii*, based on a combination of characters: type of vegetative reproduction, morphology of underground organs, cross-section of the basal leaf, and structural morphology of the shoot. It should be noted that the type of vegetative reproduction can also be effectively applied in the classification of other bulbous plants. For example, a recently described tulip species from Kazakhstan — *Tulipa jansii* J.J. de Groot & Zonn. — differs from the closely related *Tulipa kolpakowskiana* Regel primarily by the presence of stolons on its bulbs [42].

### Identification key for species similar to *Gagea kotuchovii* occurring in Asia

1. Plants in the early stages of ontogenesis with one or several long stolons2

— Plants in the early stages of ontogenesis with a short or poorly developed stolon9

1. Juvenile and young generative plants form a group of stolons with a single bulb at the end3

— Juvenile specimens form one stolon with one or several bulbs at the end7

1. Basal leaf without a cavity; seeds flat or cylindrical4

— Basal leaf tubular; seeds flat6

1. Juvenile (and rarely generative) plants form a group of long stolons that differ slightly in length; seeds flat5

— Juvenile plants form 2–10 stolons of varying lengths (5–80 mm) with bulbs densely covered with hair-like papillae; seeds cylindrical*G. kotuchovii*

1. Juvenile plants form up to 10 stolons with a bulb at the end, without their own roots; basal leaf entirely or partially with a well-developed cavity*G. nabievii*

— Juvenile and early generative plants form up to 5 stolons with a bulb, tightly enveloped by short sclerenchymatous roots; basal leaf flat-grooved, without a cavity*G. ludmilae*

1. Two basal leaves: the first — three-angled, tubular; the second — grass-like, without a cavity; inflorescence umbellate. In juvenile stage, 1–3 stolons are formed, in generative stage – one stolon with single bulbs, loosely surrounded by sclerenchymatous roots*G. sarmentosa*

— One basal leaf, grooved-concave, with ribs on the lower side, tubular; inflorescence alternate; in generative stage – up to 5 long stolons with single bulbs, loosely wrapped with sclerenchymatous roots; style deeply three-lobed*G. chinensis*

1. Plant with a large bulb at the end of the stolon and several smaller ones above it8

— Plants with a single bulb at the end of the stolon*G. praemixta*

1. Basal leaf linear, inversely wedge-shaped, noticeably expanded under the apex into a cap-like structure with an apical point; scape five-angled, with a central cavity*G. calyptrifolia*

— Basal leaf narrowly linear, gradually narrowing towards the apex; scape four-angled, without a cavity*G. angelae*

1. Stolon short (up to 10 mm), but clearly expressed10

— Stolon very short, visible in overwintered bulbs as a protrusion (fragment) 1–2 mm long11

1. Single bulb; scape with a central cavity, and when bulb formation continues – with a second lateral channel; basal leaf in cross-section tubular, three-angled-grooved*G. capusii*

— A group of bulbs of different sizes; basal leaf without a cavity, in cross-section flat-angled*G. brevistolonifera*

1. Juvenile plants — one bulb; immature and young generative plants – a few bulbs in a group12

— Vegetative bulb always singular14

1. Scape without vertical channels; vegetative bulbs coarsely cellular-13

— Scape with 1–3 vertical channels*G. shmakoviana*

1. Basal leaf inversely wedge-shaped, most expanded in the upper quarter, slightly exceeding the inflorescence; inflorescence 4–5 times shorter than the scape*G. longiscapa*

- Basal leaf narrowly linear, gradually narrowing towards the apex, in cross-section flat-angled; juvenile stage — one bulb, immature stage — a group of bulbs; inflorescence slightly shorter than the scape*G. xiphoidea*

1. Basal leaf linear, in cross-section flat-grooved, with three ribs on the lower side15

— Basal leaf in cross-section tubular, triangular, deeply grooved*G. pedata*

1. Vegetative bulb smooth; after flowering, pedicels bend downwards16

— Vegetative bulb areolate-pitted; after flowering, pedicels point upwards17

1. Plant of plains and foothills; blooms as one of the first*G. turkestanica*

— Plant of the subalpine zone, above the forest line*G. kuraminica*

1. Basal leaf 2–3 mm wide; mountain plants18

— Basal leaf narrowly linear, 1–1.5 mm wide*G. fedtschenkoana*

1. Vegetative bulb forms only in the juvenile stage; leaves and pedicels form a whorl*G. almaatensis*

— Vegetative bulb (small — in the juvenile stage, large — at the onset of flowering); above it in the scape is a vertical channel, ending in the axil of the lower subtending leaf, noticeably distant from the whorl; below is the fused base of the second leaf of the candelabrum-like inflorescence*G. huochengensis*

## Supporting information

S1 Table *Gagea* species collected for DNA extraction and their NCBI accession numbers. (DOCX)

S1 Fig Isotype of *Gagea kotuchovii* preserved in the LE herbarium. (DOCX)

S1 File Inclusivity in global research. (DOCX)
