# Correction: *Gagea kotuchovii* (Liliaceae) a new species from the Karatau Mountains (western Tian Shan, Kazakhstan) evidenced by morphological and molecular analyses

**DOI:** 10.1371/journal.pone.0345655

**Published:** 2026-03-23

**Authors:** Serik Kubentayev, Igor Levichev, Shukherdorj Baasanmunkh, Ewelina Klichowska, Daniyar Alibekov, Hyeok Jae Choi, Marcin Nobis

Fig 3 was uploaded incorrectly. Please see the correct Fig 3 here.

**Fig 3:**
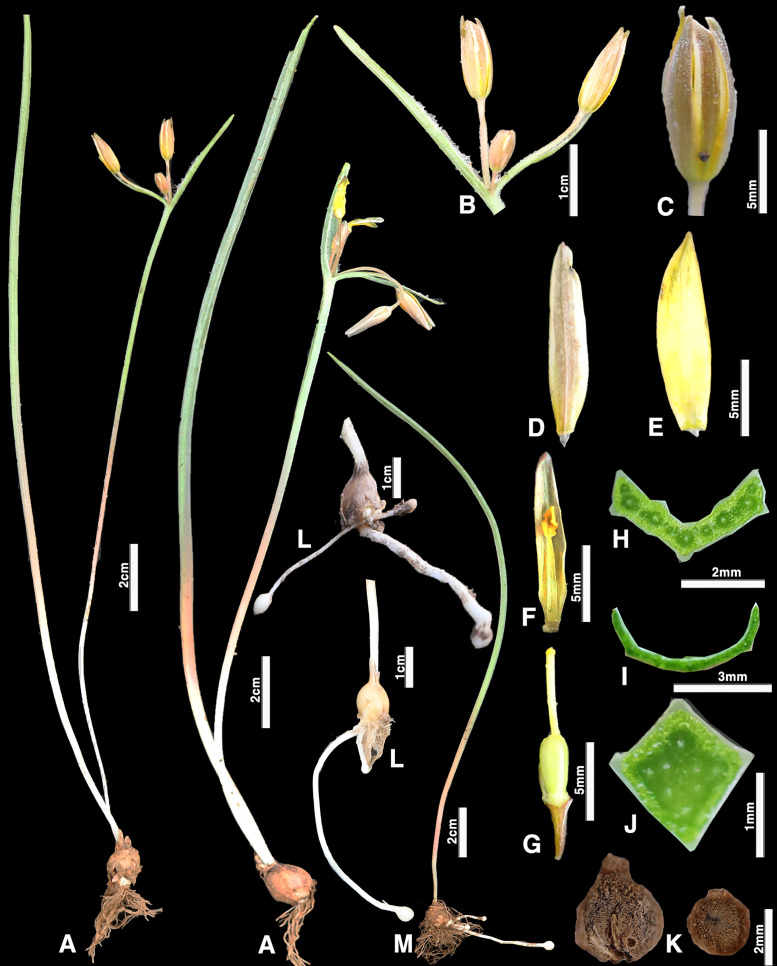
*Gagea kotuchovii*;(A) General habit; (B) inflorescence; (C) flower; (D) outer petal of the perianth; (E) inner petal of the perianth (F) perianth petal with a stamen; (G) gynoecium; (H) basal leaf transverse section; (J) peduncle cross-section; (I) stem leaf transverse section; (K) last year’s juvenile bulb; (L) bulb of a vegetative individual with several stolons; (M) vegetative individual (Photo by: S. Kubentayev).

## References

[1] Kubentayev S, Levichev I, Baasanmunkh S, Klichowska E, Alibekov D, Choi HJ, et al. *Gagea kotuchovii* (Liliaceae) a new species from the Karatau Mountains (western Tian Shan, Kazakhstan) evidenced by morphological and molecular analyses. PLoS One. 2025;20(12):e0336223. doi: 10.1371/journal.pone.0336223 41348920 PMC12680317

