# Supplementary material for: Gagea kotuchovii (Liliaceae) a new species from the Karatau Mountains (western Tian Shan, Kazakhstan) evidenced by morphological and molecular analyses
Source: PLoS One. 2025 Dec 5;20(12):e0336223. doi: 10.1371/journal.pone.0336223 (PMC12680317; doi:10.1371/journal.pone.0336223)
Supplement: S1 Table — (DOCX) [file pone.0336223.s001.docx]

**Table S1.** *Gagea* species collected for DNA extraction and their NCBI accession numbers

| **NCBI accession numbers** | **Scientific name** | **Locality** | **latitude** | **longitude** | **Collection date** | **Collector** |
| --- | --- | --- | --- | --- | --- | --- |
| PV565372 | *Gagea brevistolonifera* Levichev | Kazakhstan. Zhambyl Region, Merke district. Merke Gorge | 42.742277 | 73.228688 | 2023-04-11 | Kubentayev S.A., Alibekov D. Т. |
| PV565373 | *Gagea turkestanica* Pascher | Kazakhstan. Turkestan Region, Tyulkubasskiy district. The vicinity of the cordon outside the Aksu-Zhabagli Nature Reserve | 42.332423 | 70.370391 | 2024-04-10 | Kubentayev S.A., Alibekov D. Т. |
| PV565374 | *Gagea kotuchovii* Kubentayev et Levichev sp. nov. | Kazakhstan, Turkestan Region, near Khantagi | 43.542609 | 68.670431 | 2024-04-08 | Kubentayev S.A., Alibekov D. Т. |
