# Supplementary figures and images for: Gagea kotuchovii (Liliaceae) a new species from the Karatau Mountains (western Tian Shan, Kazakhstan) evidenced by morphological and molecular analyses
Source: PLoS One. 2025 Dec 5;20(12):e0336223. doi: 10.1371/journal.pone.0336223 (PMC12680317; doi:10.1371/journal.pone.0336223)

**Figure S1.** Isotypeof *Gagea kotuchovii* preserved in the LE herbarium

**
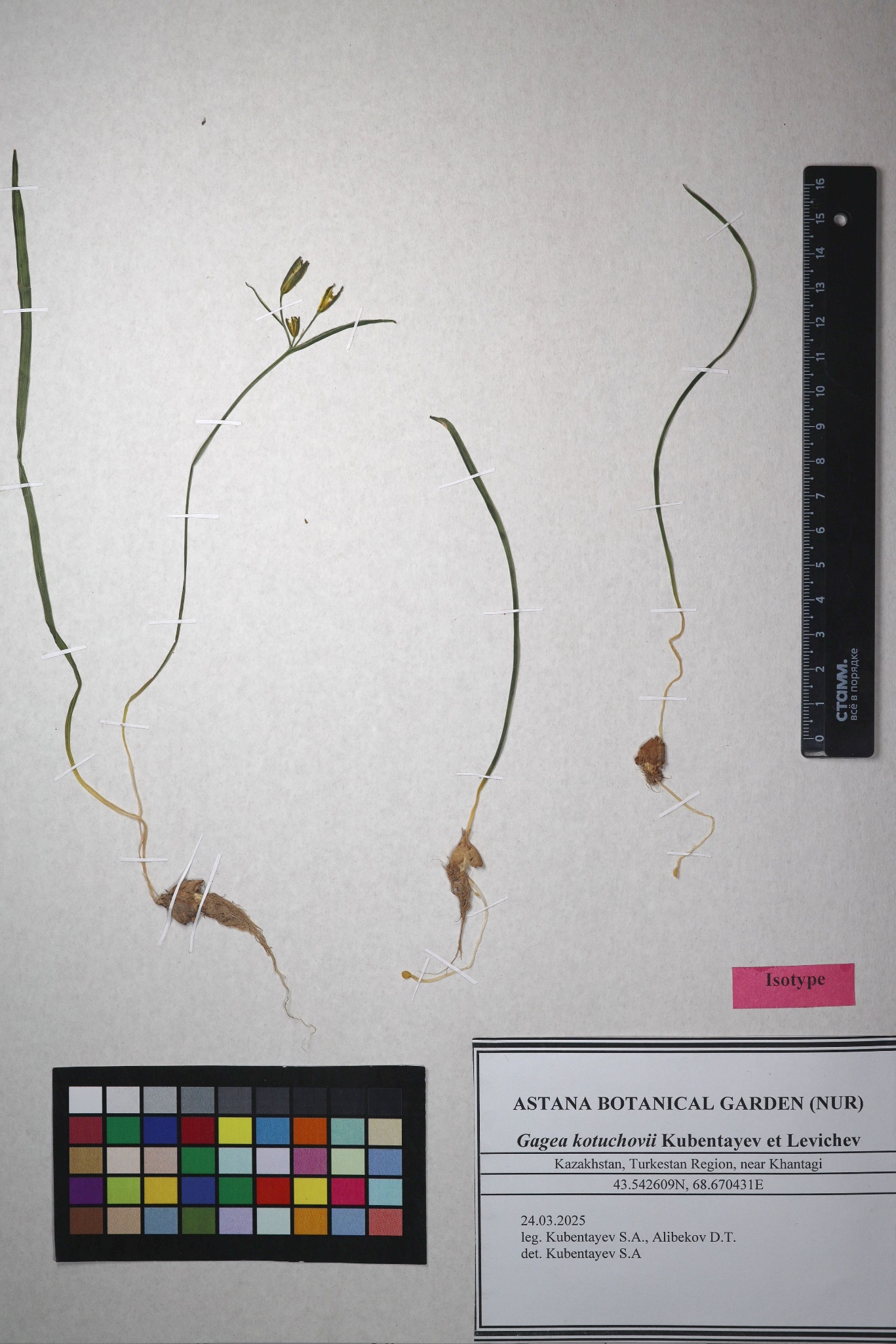
**

Supplement: S1 Fig — (DOCX) [file pone.0336223.s002.docx]
