# Supplementary material for: Gagea kotuchovii (Liliaceae) a new species from the Karatau Mountains (western Tian Shan, Kazakhstan) evidenced by morphological and molecular analyses
Source: PLoS One. 2025 Dec 5;20(12):e0336223. doi: 10.1371/journal.pone.0336223 (PMC12680317; doi:10.1371/journal.pone.0336223)
Supplement: S1 File — (DOCX) [file pone.0336223.s003.docx]

**lusivity in global research**

**Ethical considerations, permits and authorship**

Field collection was conducted in accordance with local regulations. The collection site is not legally protected, and the species is not listed as endangered. Therefore, no permits were required.

**Did this study involve local collaborators that are residents of the country where the research was conducted or members of the community studied?**

Yes, two of the authors — Serik Kubentayev (first author) and Daniyar Alibekov — are citizens of Kazakhstan, where the research was conducted and the material was collected. The authors listed above reside and conduct research in Kazakhstan.

**Human subjects research (e.g. health research, medical research, cross-cultural psychology)**

No human subjects were involved in this study.

**How did members of the local community provide input on the aims of the research investigation, its methodology, and its anticipated outcome(s)?**

Kubentayev S.A. and Alibekov D.T. conducted field research and collected material specimens. In addition, Kubentayev S.A. contributed to: Conceptualization, Data curation, Formal analysis, Investigation, Methodology, Resources, Writing – original draft.

**When engaging with the local community, how did you ensure that the informed consent documents and other materials could be understood by local stakeholders?**

This issue was not relevant to our research, as all necessary discussions with the local community were conducted in Kazakh by the first and lead author.

**Will the findings of the research be made available in an understandable format to stakeholders in the community where the study was conducted (e.g. via a presentation, summary report, copies of publications, etc.)? Please provide details of how this will be achieved.**

In this study, the collection of material and fieldwork within the country (Kazakhstan) was carried out by the first author (Serik Kubentaev) and one of the co-authors (Daniyar Alibekov), who are citizens of this country and permanent residents there. After publication of the article, all interested parties will receive copies of the publication. If necessary, the text of the article will be translated into Kazakh by the first author.

**Non-human subjects research using specimens / animals collected as part of the study, or those housed in archival collections. Examples include archaeology, paleontology, botany and zoology.**

Field collection was conducted in accordance with local regulations. The collection site is not legally protected, and the species is not listed as endangered. Therefore, no permits were required.

**If the material used in your study was imported, please A) provide the year it was imported and B) indicate whether permits were obtained to import/export the materials used, C) provide details of any permits obtained.**

No imported material was used in this study.

**If you used archival specimens, please state how the material used in your study was acquired by the institute it is held in and provide details of any permits obtained for the original excavations/ sample collection.**

No archival specimens were used in this study.

**How was the potential cultural significance of the materials collected in your study to local communities considered in your research design? Were Indigenous peoples and/or local researchers and institutions involved with archaeological excavations / collection of specimens? If so, please provide a description of their involvement.**

The materials (plant specimens) collected in this study have no cultural significance.

**If your manuscript includes photographs of human remains please indicate whether authors obtained permission from descendants or affiliated cultural communities to do so.**

The manuscript does not include photographs of human remains.
